# Receiver operating characteristic analysis using a novel combined thermal and ultrasound imaging for assessment of disease activity in rheumatoid arthritis

**DOI:** 10.1038/s41598-022-26728-4

**Published:** 2022-12-21

**Authors:** York Kiat Tan, Cassandra Hong, HuiHua Li, John Carson Allen, Julian Thumboo

**Affiliations:** 1 Department of Rheumatology and Immunology, Singapore General Hospital, Outram Road, Singapore, 169608 Singapore; 2 Duke-NUS Medical School, Singapore, Singapore; 3 Yong Loo Lin School of Medicine, National University of Singapore, Singapore, Singapore; 4 Health Services Research Unit, Singapore General Hospital, Singapore, Singapore; 5 Centre for Quantitative Medicine, Duke-NUS Medical School, Singapore, Singapore

**Keywords:** Rheumatology, Musculoskeletal system, Rheumatic diseases, Medical research, Translational research

## Abstract

We aim to determine whether combined thermal and ultrasound (CTUS) imaging can identify rheumatoid arthritis (RA) patients with at least moderate disease activity (DAS28 > 3.2). Temperature differences of maximum (T_max_), average (T_avg_) and minimum (T_min_) temperatures from a control temperature at 22 joints (bilateral hands) were summed up to derive the respective MAX, AVG and MIN per patient. MAX (PD), AVG (PD) and MIN (PD) are CTUS results derived by multiplying MAX, AVG and MIN by a factor of 2 when a patient’s total ultrasound power Doppler (PD) joint inflammation score > median score, which otherwise remained unchanged. Receiver operating characteristic (ROC) analysis was used to determine whether CTUS imaging can identify patients with DAS28 > 3.2. In this cross-sectional study, 814 joints were imaged among 37 RA patients (mean disease duration, 31 months). CTUS (but not single modality) imaging parameters were all significantly greater comparing patients with DAS28 > 3.2 versus those with DAS28 ≤ 3.2 (all *P* < 0.01). Area under the ROC curves (AUCs) using cut-off levels of ≥ 94.5, ≥ 64.6 and ≥ 42.3 in identifying patients with DAS28 > 3.2 were 0.73 , 0.76 and 0.76 for MAX (PD), AVG (PD) and MIN (PD), respectively (with sensitivity ranging from 58 to 61% and specificity all 100%). The use of CTUS in detecting a greater severity of joint inflammation among patients with at least moderate disease activity (DAS28 > 3.2) appears promising and will require further validation in independent RA cohorts.

## Introduction

Thermography and ultrasonography are imaging techniques that have been applied in rheumatoid arthritis (RA) joint inflammation assessment^1–3^. Unlike conventional radiography (CR) and computer tomography (CT) imaging modalities, they do not have risk of ionizing radiation. When compared to magnetic resonance imaging (MRI), they are also relatively cheaper and easier to set up in the routine outpatient settings. Ultrasound is often regarded as being operator dependent with the concern of reproducibility, although it has been shown that the use of consensus-based scoring along with a standardized definition of joint inflammation pathology can improve its performance and reliability as an outcome measurement tool in RA^4^. Ultrasound (and MRI) has been shown to be superior to clinical examination in the detection of joint inflammation, and the European Alliance of Associations for Rheumatology (EULAR) has included in its recommendations for use of joint imaging in the clinical management of RA that both ultrasound and MRI should be considered for more accurate assessment of inflammation^5^. Thermography relies on emitted infrared radiation generated by heat to visualize surface temperature variations and can help provide an objective assessment of the surface temperature over the target joints^6^. This is of relevance in RA, as “calor” (or heat) is one of the four classic signs of inflammation which may be encountered in an inflamed joint^7^. Modern imaging tools may afford a more precise way to assess joint inflammation thereby improving monitoring of disease activity in patients with rheumatoid arthritis (RA) which ultimately serves to guide the clinicians in making treatment decisions. As both thermography and ultrasonography represent low cost, safe, portable non-invasive imaging technologies with high feasibility for use, there has been recent interest in their combined use in detecting joint inflammation in RA. A novel combined thermal and ultrasound (CTUS) imaging approach in RA had demonstrated superiority over either imaging modality alone in terms of correlation with the routinely utilized disease activity score at 28 joints (DAS28)^8^. Combining the two imaging techniques may help complement each other^8^. A possible explanation for this may be that combining the two imaging modality has provided a more comprehensive assessment of joint inflammation and hence disease activity in patients with RA. It is well accepted that an increased synovial power Doppler (PD) vascularity on ultrasonography indicates a more active joint inflammation in RA^4^. However, apart from hypervascularization, the synovial inflammation in RA also consists of a cellular component. Various immunological cells (e.g. B cells and T cells) and the orchestrated interaction of pro-inflammatory cytokines have been implicated in the pathophysiology of RA. Synovitis occurs with new blood vessels formation along with influx and/or local activation of mononuclear cells. The resultant pannus formation can lead to bone erosion while enzymes secreted by chondrocytes and synoviocytes can cause cartilage degradation^9,10^. The increased temperature over an inflamed joint may therefore be due to (1) hyperaemia whereby the degree of synovial vascularity can be assessed using PD imaging and (2) cellular inflammation (e.g. the component from the activities of various immunological cells and the interplay of various cytokines and inflammatory mediators)—although ultrasound detects the pannus, we do not know how active the cellular component may be in the thickened synovium. This is where an objective measure of temperature by thermography may provide an additional dimension.

Although the CTUS appears promising^8^, nonetheless, there remains important practical issues that need to be addressed, one of which is to determine the performance of the CTUS in differentiating RA patients in various disease activity states, an area that has not been specifically looked into previously. In this study, we aim to further evaluate the performance of CTUS imaging in identifying RA patients with at least moderate disease activity (DAS28 > 3.2) through the use of receiver operating characteristic (ROC) analysis. DAS28 was utilized as a clinical outcome measure as it is a validated composite disease activity score commonly used in the RA clinical and trials settings^11^. DAS28 > 3.2 was chosen in our study as clinicians aim to attain the target of disease remission or low disease activity for RA patients^12^.

## Methods

This study was approved by the local institutional review board, conforming to the relevant research ethnical guidelines. In this cross-sectional study, RA patients with at least one swollen and/or tender joint(s) were recruited consecutively from the period January to November 2018 after providing their informed consent. This present study was based on the same patient cohort in our previous study^8^.

### Patients’ baseline demographics and characteristics

Baseline patient characteristics including the age, ethnicity, gender, DAS28 score, disease duration, disease modifying anti-rheumatic drugs (DMARDs) and corticosteroid use at the time of enrolment were obtained from the hospital medical records^8^.

### Thermal imaging

Thermal imaging followed the protocol in our previous study^8^. Using established methods in the literature^13–15^, thermography data was prospectively collected and carried out by designated research staff in the same windowless (draft-free) room with a controlled room temperature of about 22 °C^13^. The patients were rested for 15 min prior to the start of thermal imaging to allow for acclimatization as per standard practice^13^. To standardize the imaging procedure, the dorsal view of each patient’s bilateral hands was sequentially placed in a neutral position on a flat table top and separately imaged with the thermal camera situated 50 cm directly above the hand. Physical objects (such as watches) which can obscure the view of the thermal camera had to be removed. Using a regions of Interests (ROIs) manual segmentation approach^14^, rectangular boxes were placed over each target joint site. The target joint sites included the bilateral wrists and the following small joints of the digits bilaterally: metacarpophalangeal joints (MCPJs), thumb interphalangeal joints (IPJs) and proximal interphalangeal joints (PIPJs). A portable FLIR T640 high performance thermal camera (specifications: thermal sensitivity of < 30 milli-Kelvin (mK) at 30 °C, 640 × 480 pixel resolution and predefined emissivity value of 0.98 for skin^15^) was utilized for thermography. The following temperature measurements (utilized in previously published studies^10,14,15^) were recorded in °C at each target joint site: maximum temperature (T_max_), minimum temperature (T_min_) and average temperature (T_avg_). Temperature differences of T_max_, T_avg_ and T_min_ from a control temperature at 22 joints (bilateral hands) were summed up to derive the respective MAX, AVG and MIN for each patient. The control temperature (specific for each individual patient) was defined as the lowest T_min_ temperature among all joints testing negative for both ultrasound PD and grey-scale (GS) joint inflammation^3,16^.

### Ultrasound imaging

Ultrasound imaging followed the protocol in our previous study^8^. Standardized ultrasound scanning based on the EULAR guidelines^17^ was carried out on the same day as the thermography during each patient’s study visit. Ultrasound scans were acquired prospectively and scored by a single rheumatologist (blinded to the thermal imaging results) experienced in musculoskeletal ultrasonography using the Mindray M9 ultrasound machine with a L14-6Ns linear probe, while a separate trained study team member performed the thermal imaging. The joint sites of the bilateral hands scanned at the dorsal recesses by ultrasound were as follows: wrists at the (a) radiocarpal, intercarpal joints and (b) distal radioulnar joint; 1st to 5th MCPJs (from thumb to little finger); thumbs’ IPJs; 2nd to 5th PIPJs (from index to little finger). Ultrasonography was performed at the joints with the following pre-sets: pulse repetition frequency at 900 Hz; Doppler frequency at 5 MHz. For ultrasound scoring, GS and PD joint inflammation were graded semi-quantitatively at each joint recess using a 0–3 severity scale (none = 0; mild = 1; moderate = 2; severe = 3) using previously validated scoring methods shown to have acceptable inter/intra-observer reliability^18,19^. Specifically, an ultrasonographic atlas were utilized for ultrasound GS joint inflammation scoring^18^, while the definitions of Backhaus et al. were utilized for ultrasound PD joint inflammation scoring^19^. As each finger joint involved scanning of a single joint recess, while each wrist included scanning of two joint recesses, the average of the scores at the two joint recesses was computed for both ultrasound GS and PD scoring at the wrists. The ultrasound GS and PD joint inflammation sub-scores at all the 22 joint sites of the bilateral hands were then summed up to obtain the Total PD and Total GS scores for each patient.

### Combined thermal and ultrasound imaging

MAX (PD), AVG (PD) and MIN (PD) are CTUS results derived from combining thermal imaging with ultrasound PD imaging as per our previous study^8^. MAX (PD), AVG (PD) and MIN (PD) are computed by multiplying MAX, AVG and MIN by a factor of 2 when a patient’s total ultrasound PD joint inflammation score is greater than the median score of the study population^8^. As per previously described^8^, this is performed to increase the weightage of a patient’s Total PD score to the CTUS imaging scores as a greater PD vascularity is indicative of a greater amount of inflammation at the joints; moreover, it has been well demonstrated in histopathology studies that PD signals correlate well with synovium inflammation at the joints^20,21^. In the event that the Total PD score of a patient was less than or equal to the median score of the study population, the CTUS results, i.e. MAX (PD), AVG (PD) and MIN (PD), remained unchanged from the MAX, AVG and MIN (without multiplying by a factor of 2). We arbitrarily adopted the median total ultrasound PD joint inflammation score (i.e. the 50th percentile) of the study population as a cut-off in deriving a patient’s MAX (PD), MIN (PD) and AVG (PD), as currently there is no universal consensus on what constitutes a low or high inflammatory burden on ultrasound imaging in RA at the patient level.

### Statistical analysis

For both CTUS and single modality imaging parameters (MAX (PD), AVG (PD) and MIN (PD), MAX, AVG, MIN, Total GS score and Total PD score), their mean results were compared between (a) patients with DAS28 ≤ 3.2 and (b) patients with DAS28 > 3.2 using the 2-sample t-test. For imaging parameter(s) found to be significantly different between the two DAS28 patient groups, sensitivity, specificity and ROC analysis was used to further determine whether these parameter(s) can differentiate RA patients into the following two groups: those with DAS28 > 3.2 and those with DAS28 ≤ 3.2. The ‘Closest to Top Left’ method was used to determine the optimal ROC curve cut-off value. The analyses were performed using R 3.6.2 (www.r-project.org).

### Ethics approval and consent to participate

This study was approved by the SingHealth Centralised Institutional Review Board (Ref. No.: 2017/3003) and conforms to the relevant research ethnical guidelines. All recruited patients provided their informed consent prior to enrolment.

## Results

### Patients’ baseline characteristics

Our patients’ baseline characteristics have been described previously^8^ and will be briefly described here. A total of 814 joints were imaged among 37 RA patients. At baseline, the mean (SD) of the age of the patients was 57 (14) years. Out of 37 patients, 28 (76%) were female and 28 (76%) were Chinese. The mean (SD) disease duration and DAS28 scores of the study population were 31 (45) months and 4.4 (1.1) respectively. At the time of recruitment, out of 37 patients, 31 (84%) were on one or more DMARDs (hydroxychloroquine, methotrexate, sulfasalazine, and/or to facitinib) and 26 (70%) were on prednisolone.

### Comparison of results from imaging parameters between DAS28 patient groups

For CTUS imaging (see Table 1), the mean MAX (PD), AVG (PD) and MIN (PD) results were all statistically significantly greater among patients with DAS28 > 3.2 versus those with DAS28 ≤ 3.2 (all *P* < 0.01). In contrast, for single modality imaging (see Table 1), the mean MAX. AVG, MIN, Total PD score and Total GS score were all not statistically significantly different between patients with DAS28 > 3.2 and those with DAS28 ≤ 3.2 (all *P* > 0.05).

**Table 1:** Comparison of results from imaging parameters between DAS28 patient groups.

Imaging parameter	Mean (95% CI)			*P*-value
	Group A: DAS28 ≤ 3.2 (n = 6)	Group B: DAS28 > 3.2 (n = 31)	Difference between groups A and B	
Thermography alone^a^				
MAX	67 (51, 84)	82 (75, 90)	−15 (−34, 4)	0.13
AVG	45 (34, 57)	59 (53, 65)	−14 (−29, 2)	0.08
MIN	30 (22, 38)	40 (35, 45)	−10 (−22, 3)	0.12
Ultrasound alone				
Total GS	6.7 (1.3, 12.0)	6.6 (4.1, 9.0)	0.1 (−6.2, 6.4)	0.98
Total PD	2.8 (−0.2, 5.9)	3.7 (2.7, 4.6)	−0.8 (−3.4, 1.8)	0.53
CTUS^b^				
MAX (PD)	75 (59, 92)	120 (101, 138)	−44 (−70, −18)	*P* < 0.01**
AVG (PD)	51 (39, 62)	85 (72, 99)	−35 (−53, −16)	*P* < 0.001***
MIN (PD)	34 (26, 42)	58 (48, 67)	−24 (−37, −10)	*P* < 0.01**

*GS* grey-scale, *PD* power Doppler, *CTUS* combined thermal and ultrasound.

^a^MAX, AVG and MIN are thermographic parameters derived from summing the temperature differences between the respective maximum (T_max_), average (T_avg_) and minimum (T_min_) temperatures at the joints with a control temperature for each patient

^b^MAX (PD), AVG (PD) and MIN (PD) are combined thermal and ultrasound imaging results derived by multiplying MAX, AVG and MIN by a factor of 2 when a patient’s total ultrasound power Doppler joint inflammation score exceeds the median score. Otherwise, MAX (PD), AVG (PD) and MIN (PD), remained unchanged from the MAX, AVG and MIN.

Statistically significant: **P < 0.01, ***P < 0.001.

### ROC analysis

The area under the ROC curves (AUCs) (95%CI) using optimal cut-off levels of ≥ 94.5, ≥ 64.6 and ≥ 42.3 in identifying patients with DAS28 > 3.2 were 0.73 (0.54, 0.92), 0.76 (0.60, 0.93) and 0.76 (0.59, 0.93) for MAX (PD), AVG (PD) and MIN (PD), respectively (see Table 2) with sensitivity ranging from 58 to 61% and specificity all 100% (the specific sensitivity, specificity, negative predictive value, positive predictive value and accuracy results for the CTUS imaging parameters are summarized in Table 2).

**Table 2:** Use of CTUS imaging in identifying RA patients with DAS28 > 3.2 applying ROC analysis.

Imaging parameter^a^	Cut-off levels	AUC (95% CI)	Sn (%)	Sp (%)	NPV (%)	PPV (%)	Accuracy (%)
MAX (PD)	94.5	0.73 (0.54, 0.92)	58	100	32	100	65
AVG (PD)	64.6	0.76 (0.60, 0.93)	61	100	33	100	68
MIN (PD)	42.3	0.76 (0.59, 0.93)	61	100	33	100	68

AUC, area under the ROC curves; Sn, sensitivity; Sp, specificity; NPV, negative predictive value; PPV, positive predictive value.

^a^MAX (PD), AVG (PD) and MIN (PD) are combined thermal and ultrasound imaging results derived by multiplying MAX, AVG and MIN by a factor of 2 when a patient’s total ultrasound power Doppler joint inflammation score exceeds the median score. Otherwise, MAX (PD), AVG (PD) and MIN (PD), remained unchanged from the MAX, AVG and MIN.

## Discussion

Despite efforts to delineate the role of musculoskeletal imaging in RA joint inflammation assessment, there remains important practical issues that need to be addressed, one of which is to determine the performance of musculoskeletal imaging tools as biomarkers to help differentiate RA patients in various disease activity states. This is important as modern imaging tools may afford a more precise way to assess joint inflammation thereby improving monitoring of disease activity in patients with RA which ultimately serves to guide the clinicians in making treatment decisions for their patients. We have therefore added to the RA literature through our new findings from this current study that the CTUS imaging approach can help detect a greater severity of joint inflammation among RA patients with at least moderate disease activity (DAS28 > 3.2). Through the use of Receiver Operating Characteristic (ROC) analysis, we have further derived the optimal cut-off results for CTUS parameters in identifying patients with at least moderate disease activity (DAS28 > 3.2). The ability to differentiate RA patients based on DAS28 results has practical implications as clinicians aim to attain the treatment target of disease remission or low disease activity (i.e. targeting DAS28 to ≤ 3.2) for RA patients.

A common effective approach to assess the performance of a diagnostic test is to utilize ROC analysis^22,23^ which may help derive optimum cut-off values to discriminate between clinical disease states. For ultrasound, two previous studies^24,25^ have investigated the use of ultrasound PD and GS joint inflammation in studying disease activity states in patients with RA by applying ROC analysis. In the first study by Leng et al.^24^, 82 RA patients on infliximab were followed up over 22 weeks. The clinically dominant hand were assessed by ultrasound at the following joint sites: wrist; 2nd and 3rd MCPJs; 2nd and 3rd PIPJs; 2nd and 5th MTPJs. Ultrasound GS and PD synovitis were graded semi-quantitatively using a 0–3 severity scale while GS tenosynovitis/paratendinitis were graded using a binary system (present = 1 or absent = 0). Utilizing ROC analysis, the AUC results using cut-offs of less than 29 for GS score and less than 1 for PD scores for determination of RA disease remission (defined as DAS28 < 2.6) at 22 weeks were 0.6501, and 0.7474, respectively. Next, in a cross-sectional study by Li et al.^25^ involving 30 RA subjects using an extended 36 joint sites ultrasonography (involving the bilateral hands, feet, elbows, and ankles), ultrasound joint inflammation was scored semi-quantitatively using a 0 to 3 severity scale. The AUC based on cut-off PD scores ≥ 2.5 for identifying those with high disease activity (DAS28 > 5.1) was 0.88 (with sensitivity = 100%, specificity = 69.2%, accuracy = 73.3%). For thermography, there has been limited data about its use in differentiating RA patients based on their disease activity states and studies have mostly focuses on detecting differences between RA patients and non-RA subjects (e.g. healthy controls)^26–28^. A recent study by Pauk et al.^29^ evaluated the use of dynamic thermography at the five digits of a hand in 66 RA patients (50 patients had high disease activity with DAS28 > 5.1; 16 patients had moderate disease activity with 5.1 > DAS28 > 3.2) and 42 healthy participants although ROC analysis was not specifically performed. Dynamic data from three video frames (before cooling, post-cooling, and post-rewarming) were acquired with the following observations made from the study: (a) a lower area under the heating curve, (b) a lower difference between the area under the rewarming curve and the cooling curve and (c) a smaller total change in mean temperature due to rewarming were found among RA patients with high disease activity versus those with moderate disease activity (*p* < 0.05). The three studies mentioned above by Leng et al.^24^, Li et al.^25^ and Paul et al.^29^ has applied either ultrasound or thermal imaging to detect differences in imaging outcomes between various DAS28 patient groups as defined in their respective studies, while our current study have added to the RA literature by evaluating the performance of a novel combined thermal and ultrasound imaging approach in identifying RA patients with at least moderate disease activity (DAS28 > 3.2). In our current CTUS imaging study, we did not utilize dynamic thermography. Future RA studies can evaluate whether there may be any important differences when thermographic parameters are derived from dynamic versus static thermography.

Two other findings from our current study deserve to be mentioned. First, the results from single modality imaging parameters in our study were not significantly different in those patients with DAS28 > 3.2 versus those with DAS28 ≤ 3.2 (all *P* > 0.05). Second, although we have achieved high specificity results (all 100%) for the derived cut-off levels for PD MAX (PD), AVG (PD) and MIN (PD), the sensitivity results were relatively lower, ranging from 58 to 61%. One possible explanation could be that in our study, only joint sites from bilateral hands were included. Previous RA studies have shown that an extended ultrasonography at 36 joint sites (including bilateral hands, feet, elbows and ankles) correlated significantly with DAS28 (r = 0.46, *p* < 0.05)^30^ and patients with ultrasound PD synovitis positivity had significantly higher DAS28 scores when compared to those with PD negativity (*p* < 0.05) with a sensitivity of 100% attained (specificity was lower at 69.2%) when a cut-off PD score ≥ 2.5 was used to identify RA patients with high disease activity (DAS28 > 5.1)^25^. Future studies should look into whether there may be any minimum and/or optimal number of joint sites and whether the specific locations of the joint sites selected may influence the performance of these imaging modalities when used as combined or individual imaging tools.

Our study has its limitations. Apart from the relatively small sample size, we have only imaged the bilateral hand joints although the wrists and the small joints of the hands (e.g. MCPJs and PIPJs) are among the most commonly affected joint sites in patients with RA^31–33^. In our study, both thermal and ultrasound imaging were studied in relation to patients’ RA disease activity states at a single time-point using a cross-sectional study design. Our study cohort also has an overall longstanding disease duration with patients only on one or more of the following DMARDs: hydroxychloroquine, methotrexate, sulfasalazine, and/or tofacitinib. Therefore, the performance of our CTUS imaging will need to be further tested out in larger RA cohorts using different combinations of joint sites. Our CTUS approach will also need to be tested out longitudinally with imaging performed over multiple time points and evaluated among patients with diverse clinical and treatment profiles. In our present study, we have utilized the median total ultrasound PD joint inflammation score (i.e. the 50th percentile) of the study population as a cut-off in deriving a patient’s MAX (PD), MIN (PD) and AVG (PD). Future larger scale studies can investigate the use of various methods (apart from using the median total ultrasound PD joint inflammation score) to define the cut-off(s) in deriving the CTUS imaging results. For the median total ultrasound PD joint inflammation score, it is possible that a different value of “population median” may exist in another RA cohort with a different patient profile (e.g. in early RA disease), although further studies will be necessary to help investigate and clarify this aspect. Our derived cut-off values from the ROC analysis will need to be further validated in an external, independent dataset before they can be more widely used. In our study, we have utilized static thermography and hence the utility of dynamic versus static thermography will also need to be further clarified. The “heat” component of joint inflammation in RA and how it may relate to processes of increased vascularity, activities of various immunological cells and the interplay of various cytokines and inflammatory mediators in the inflamed synovium have not been well studied. This aspect will need to be examined by well-designed histopathology RA studies in relation to thermal imaging.

## Conclusion

In summary, we have added to the RA literature through our new findings from this current study that a novel CTUS imaging approach can help detect a greater severity of joint inflammation among patients with at least moderate disease activity (DAS28 > 3.2). Through the use of ROC analysis, we have derived the cut-off MAX (PD), MIN (PD) and AVG (PD) levels for identifying patients with at least moderate disease activity (DAS28 > 3.2). The use of CTUS imaging appears promising and our findings are likely to spur further validation work in the area of disease activity assessment in RA.

## Data Availability

All data generated or analysed during this study are included in this published article and its supplementary information files.

## Abbreviations

RA: Rheumatoid arthritis
CR: Conventional radiography
CT: Computer tomography
MRI: Magnetic resonance imaging
CTUS: Combined thermal and ultrasound
DAS28: Disease activity score at 28 joints
PD: Power Doppler
ROC: Receiver operating characteristic
DMARDs: Disease modifying anti-rheumatic drugs
MCPJs: Metacarpophalangeal joints
IPJs: Interphalangeal joints
PIPJs: Proximal interphalangeal joints
ROIs: Regions of Interests
T_max_: Maximum temperature
T_min_: Minimum temperature
T_avg_: Average temperature
GS: Grey-scale
